# Supplementary material for: HIVprotI: an integrated web based platform for prediction and design of HIV proteins inhibitors
Source: J Cheminform. 2018 Mar 9;10:12. doi: 10.1186/s13321-018-0266-y (PMC5845081; doi:10.1186/s13321-018-0266-y)
Supplement: Supplementary file 2 — Additional file 2. Source code of HIVProtI web server. [file 13321_2018_266_MOESM2_ESM.zip › HIVprotI_Source-code/hivprotI/weka-3-6-6/doc/overview-summary.html]

Overview


---


|  |  |  |
| --- | --- | --- |
| **Overview** \| Package \| Class \| **Tree** \| **Deprecated** \| **Index** \| **Help** \| **Weka's home** | |  |
| PREV   NEXT | **FRAMES**    **NO FRAMES**     **All Classes** |  |


---

| **Packages** | |
| --- | --- |
| **weka.associations** |  |
| **weka.associations.gsp** |  |
| **weka.associations.tertius** |  |
| **weka.attributeSelection** |  |
| **weka.classifiers** |  |
| **weka.classifiers.bayes** |  |
| **weka.classifiers.bayes.blr** |  |
| **weka.classifiers.bayes.net** |  |
| **weka.classifiers.bayes.net.estimate** |  |
| **weka.classifiers.bayes.net.search** |  |
| **weka.classifiers.bayes.net.search.ci** |  |
| **weka.classifiers.bayes.net.search.fixed** |  |
| **weka.classifiers.bayes.net.search.global** |  |
| **weka.classifiers.bayes.net.search.local** |  |
| **weka.classifiers.evaluation** |  |
| **weka.classifiers.functions** |  |
| **weka.classifiers.functions.neural** |  |
| **weka.classifiers.functions.pace** |  |
| **weka.classifiers.functions.supportVector** |  |
| **weka.classifiers.lazy** |  |
| **weka.classifiers.lazy.kstar** |  |
| **weka.classifiers.meta** |  |
| **weka.classifiers.meta.nestedDichotomies** |  |
| **weka.classifiers.mi** |  |
| **weka.classifiers.mi.supportVector** |  |
| **weka.classifiers.misc** |  |
| **weka.classifiers.pmml.consumer** |  |
| **weka.classifiers.rules** |  |
| **weka.classifiers.rules.part** |  |
| **weka.classifiers.trees** |  |
| **weka.classifiers.trees.adtree** |  |
| **weka.classifiers.trees.ft** |  |
| **weka.classifiers.trees.j48** |  |
| **weka.classifiers.trees.lmt** |  |
| **weka.classifiers.trees.m5** |  |
| **weka.classifiers.xml** |  |
| **weka.clusterers** |  |
| **weka.clusterers.forOPTICSAndDBScan.Databases** |  |
| **weka.clusterers.forOPTICSAndDBScan.DataObjects** |  |
| **weka.clusterers.forOPTICSAndDBScan.OPTICS\_GUI** |  |
| **weka.clusterers.forOPTICSAndDBScan.Utils** |  |
| **weka.core** |  |
| **weka.core.converters** |  |
| **weka.core.logging** |  |
| **weka.core.mathematicalexpression** |  |
| **weka.core.matrix** |  |
| **weka.core.neighboursearch** |  |
| **weka.core.neighboursearch.balltrees** |  |
| **weka.core.neighboursearch.covertrees** |  |
| **weka.core.neighboursearch.kdtrees** |  |
| **weka.core.pmml** |  |
| **weka.core.stemmers** |  |
| **weka.core.tokenizers** |  |
| **weka.core.xml** |  |
| **weka.datagenerators** |  |
| **weka.datagenerators.classifiers.classification** |  |
| **weka.datagenerators.classifiers.regression** |  |
| **weka.datagenerators.clusterers** |  |
| **weka.estimators** |  |
| **weka.experiment** |  |
| **weka.experiment.xml** |  |
| **weka.filters** |  |
| **weka.filters.supervised.attribute** |  |
| **weka.filters.supervised.instance** |  |
| **weka.filters.unsupervised.attribute** |  |
| **weka.filters.unsupervised.instance** |  |
| **weka.filters.unsupervised.instance.subsetbyexpression** |  |
| **weka.gui** |  |
| **weka.gui.arffviewer** |  |
| **weka.gui.beans** |  |
| **weka.gui.beans.xml** |  |
| **weka.gui.boundaryvisualizer** |  |
| **weka.gui.experiment** |  |
| **weka.gui.explorer** |  |
| **weka.gui.graphvisualizer** |  |
| **weka.gui.hierarchyvisualizer** |  |
| **weka.gui.sql** |  |
| **weka.gui.sql.event** |  |
| **weka.gui.streams** |  |
| **weka.gui.treevisualizer** |  |
| **weka.gui.visualize** |  |
| **weka.gui.visualize.plugins** |  |

---


|  |  |  |
| --- | --- | --- |
| **Overview** \| Package \| Class \| **Tree** \| **Deprecated** \| **Index** \| **Help** \| **Weka's home** | |  |
| PREV   NEXT | **FRAMES**    **NO FRAMES**     **All Classes** |  |


---
